# Complications of therapeutic plasma exchange: A retrospective study of 1201 procedures in 435 children

**DOI:** 10.1097/MD.0000000000018308

**Published:** 2019-12-16

**Authors:** Jing Lu, Lijuan Zhang, Cheng Xia, Yuhong Tao

**Affiliations:** a Department of Pediatrics, West China Second University Hospital, Sichuan University, Chengdu; b Key Laboratory of Birth Defects and Related Diseases of Women and Children (Sichuan University), Ministry of Education; c Department of Pediatrics, People's Hospital of Ganzi Tibetan Autonomous Prefecture, Kangding, China.

**Keywords:** allergic reaction, children, complication, therapeutic plasma exchange

## Abstract

Therapeutic plasma exchange (TPE) is now widely used in therapy of multiple diseases in children, by removing the plasma with pathogenic agents from patients. However, adverse reactions may limit its application.

A retrospective cohort study of 435 hospitalized children treated with 1201 plasma exchange procedures between January 2013 and July 2018 were enrolled.

Complications occurred in 152 procedures (12.7%); 90 procedures (7.5%) had ≥2 complications. No death occurred. The most common complications were pruritus and urticaria (7%), followed by hypertension (1.92%) and hypotension (1.17%). One child had an outbreak of disseminated cryptococcosis neoformans infection, another child developed anaphylactic shock, and 3 children presented toxic epidermal necrolysis after TPE. The incidence of pruritus and urticaria was higher in children of the 6∼15 year group (*P* < .05) compared with other age groups. There was no significant difference in the incidence of hypertension and hypotension in children at different ages and weights (*P* > .05). Compared with other diseases, anti-*N*-methyl-d-aspartate (anti-NMDA) receptor encephalitis led to a higher incidence of complications in children (*P* < .05).

The results suggest that TPE is a relatively safe procedure for children, and most of the complications are mild. The most common complication is pruritus and urticaria. However, serious complications such as toxic epidermal necrolysis and infection should still be taken seriously.

## 1 Introduction

The therapeutic plasma exchange (TPE) is a therapy in which plasma is separated from the blood cellular components ex vivo, discarded and replaced with an isosmotic fluid (commonly 5% albumin or frozen plasma) to maintain appropriate oncotic pressure in the patient. In 1960, Schwab and Fahey performed the first therapeutic plasmapheresis to reduce elevated globulin level in a patient with macroglobulinemia.^[1]^ In 2016, clinical indications have expanded to 87 diseases, such as rheumatic immune diseases, autoimmune nervous system diseases, digestive system diseases, blood system diseases, kidney diseases, organ transplantation, autoimmune skin diseases, metabolic diseases, and drug poisoning.^[2]^ The goal of this therapy is to remove high-molecular-weight molecules, such as antibodies of systemic lupus erythematosus (SLE); IgA-containing immune complexes of Henoch–Schönlein purpura; circulating autoantibodies of Myasthenia gravis; IgG antibodies targeting the synaptic GluN1 subunit of the *N*-methyl d-aspartate receptor (NMDAR) of NMDAR antibody encephalitis; albumin-bound toxins as well as unbound toxins, including aromatic amino acids, ammonia, endotoxin, indols, mercaptans, phenols, and other factors which may be responsible for hepatic coma of acute liver failure patient; and drug metabolites, cytokines, or other mediators of keratinocyte cytotoxicity of toxic epidermal necrolysis and so on. TPE is currently an accepted therapy for selected indications in children.^[3]^ Even the newborns can receive TPE treatment as long as they have suitable vascular access and filters.^[4]^

Although principles of TPE are the same in adults and children, there are technical differences, such as establishment of vascular access and volume distribution. Risk of this therapy in children may be higher than that in adults.^[5]^ Although this problem has been studied in a series of studies published over the last 30 years, most are data from adults.^[6]^ The knowledge on TPE complications in children is still limited.^[7,8]^ Accordingly, we conducted a retrospective study to investigate the complications of 1201 TPE procedures and discuss the safety of TPE in children.

## 2 Methods

### 2.1 Subjects

A series of hospitalized children who underwent at least 1 TPE procedure between January 2013 and July 2018 were enrolled. Studies were conducted with the approval of the Ethics Committee of West China Second University Hospital, Sichuan University and in accordance with the Declaration of Helsinki (1951) of the World Medical Association. Written informed consent was obtained from the children's guardians before treatment.

### 2.2 TPE procedures

Treatment was performed according to the blood purification standard operating procedure of TPE in China, which included establishing appropriate vascular access for corresponding age, plasma dose prescription, anticoagulation of the circuit, and so on. Vascular access was established using the Gambro temporary single-needle double-lumen catheter (specification: 6F, 8F, or 11F) through the femoral vein. Procedures were performed with the Gambro Prismaflex (Gambro Lundia AB, Lund, Sweden) or Fresenius machine and the corresponding set: TPE1000 and TPE2000, or P1 and P2. Almost all children had anticoagulation with low-molecular-weight heparin (LMWH) of 60 to 80 IU/kg, and few children with very poor coagulation function had no anticoagulation. The blood pump flow rate was 3 to 5 mL/kg/min and does not exceed 120 mL/min. Two plasma pumps (one pumping out the patient's plasma and the other pumping in the alternative fresh frozen plasma [FFP]) were at the same speed, about 15% to 25% of the blood pump speed. Each TPE treatment will remove plasma which is approximately 5% of the child's body weight and an equal amount of FFP was used as a replacement, the total amount does not exceed 3000 mL. An extra part of 100 to 150 mL plasma was needed to prime the extracorporeal circuit to prevent insufficient cycle capacity when the child weighs >10 kg. All children received intravenous calcium supplementation to prevent hypocalcemia caused by citrate in plasma preservation solutions, the dose was 2 mL of 10% calcium gluconate solution per each 250 mL FFP. If there are symptoms of hypocalcaemia such as perioral and limb numbness, an additional 10% calcium gluconate would be added. In the course of treatment, the children were given intravenous infusion of dexamethasone (0.5 mg/kg, maximum 20 mg) to prevent allergy.

### 2.3 Data collection

Clinical data such as age, sex, weight, clinical indications, and complications were collected by nurses in the blood purification unit for children.

### 2.4 Statistics

All data were processed using SPSS 21.0 statistical software (IBM, Armonk, NY). The counting data were expressed as the composition ratio, and Pearson *χ*^2^ test was used for comparison. It was considered statistically significant when *P* < .05.

## 3 Results

### 3.1 Demographic characteristics

During 5 years, 1201 TPE treatments were performed on a total of 435 children, including 198 males (45.5%) and 237 females (54.5%). The median age was 9.16 years, with a range from 3 months to 15 years. Seventy-five patients (17.2%) were aged 3 months to 3 years, 67 patients (15.4%) were aged 3 to 6 years, and 293 patients (67.4%) were aged 6 to 15 years. The median body weight was 26 kg, with a range from 6.75 kg to 67 kg. Twenty-eight patients (6.4%) weighed ∼10 kg, 245 patients (56.3%) weighed 10∼30 kg, and 162 patients (37.2%) weighed 30∼67 kg.

### 3.2 Indications of TPE

**Figure 1:**
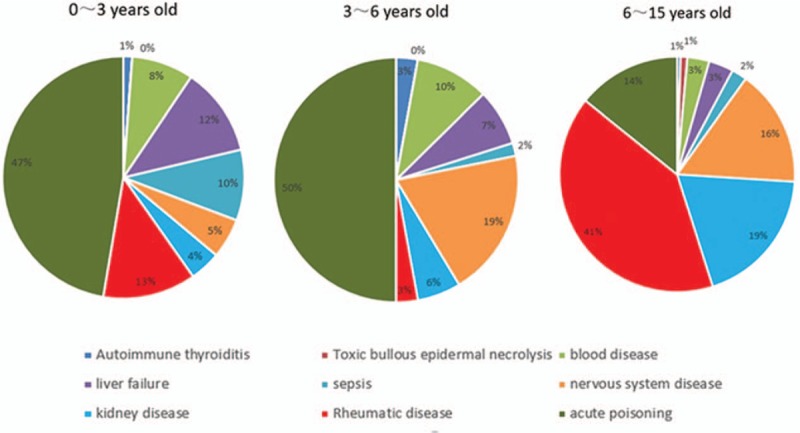
Indications of 1201 therapeutic plasma exchange procedures in 435 children of different ages.

**Figure 2:**
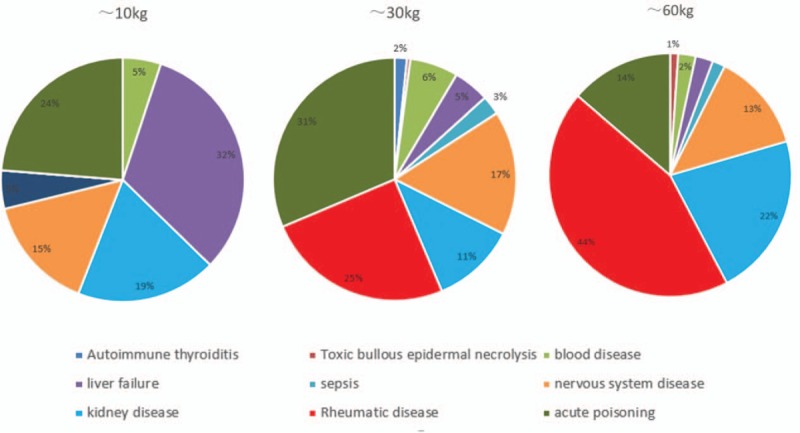
Indications of 1201 therapeutic plasma exchange procedures in 435 children at different weights.

Indications for TPE are shown in Table 1. The top 4 clinical indications were acute poisoning, rheumatic diseases, kidney diseases, and neurological diseases. The distribution of indications in children of different ages and weights is shown in Figures 1 and 2. The proportion of children with rheumatic diseases and kidney diseases was higher in the 6∼15 year group and the 30∼67 kg group.

**Table 1:**
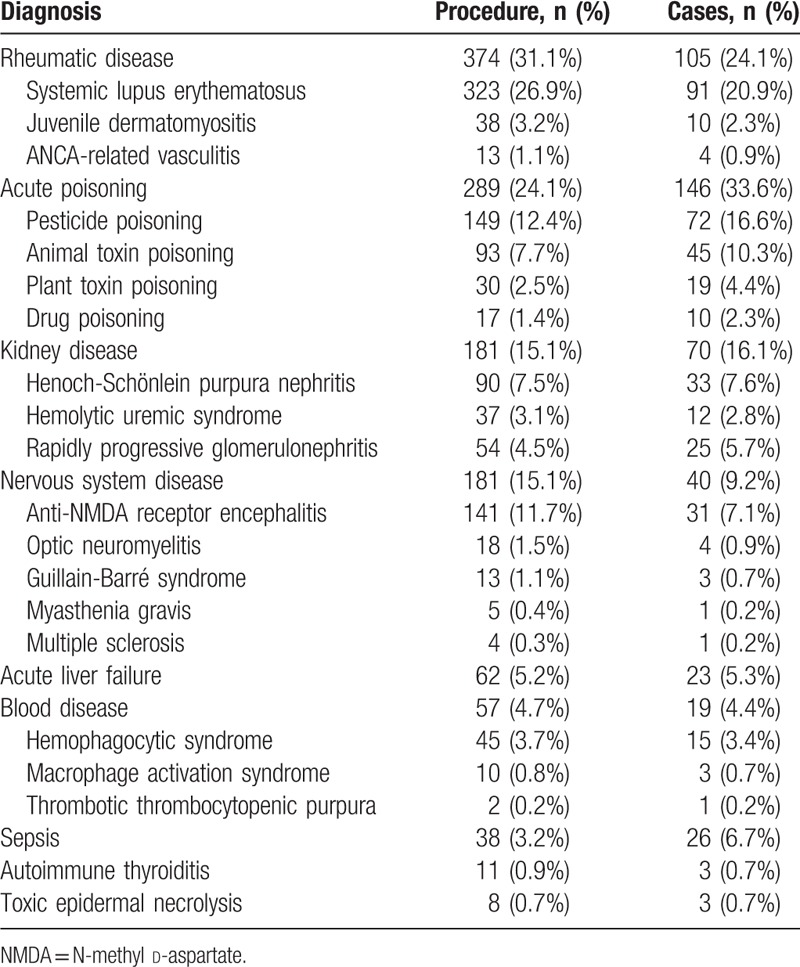
Indications of 1201 therapeutic plasma exchange procedures in 435 children.

### 3.3 Overall complications in children

Of total 1201 treatments, 152 procedures (12.7%) involved some complications. There were 90 procedures (7.5%) involving ≥2 complications during treatment. No death occurred. The overall incidence and type of complications are shown in Table 2. The most common complications were pruritus and urticaria (7%), followed by hypertension (1.92%) and hypotension (1.17%).

**Table 2:**
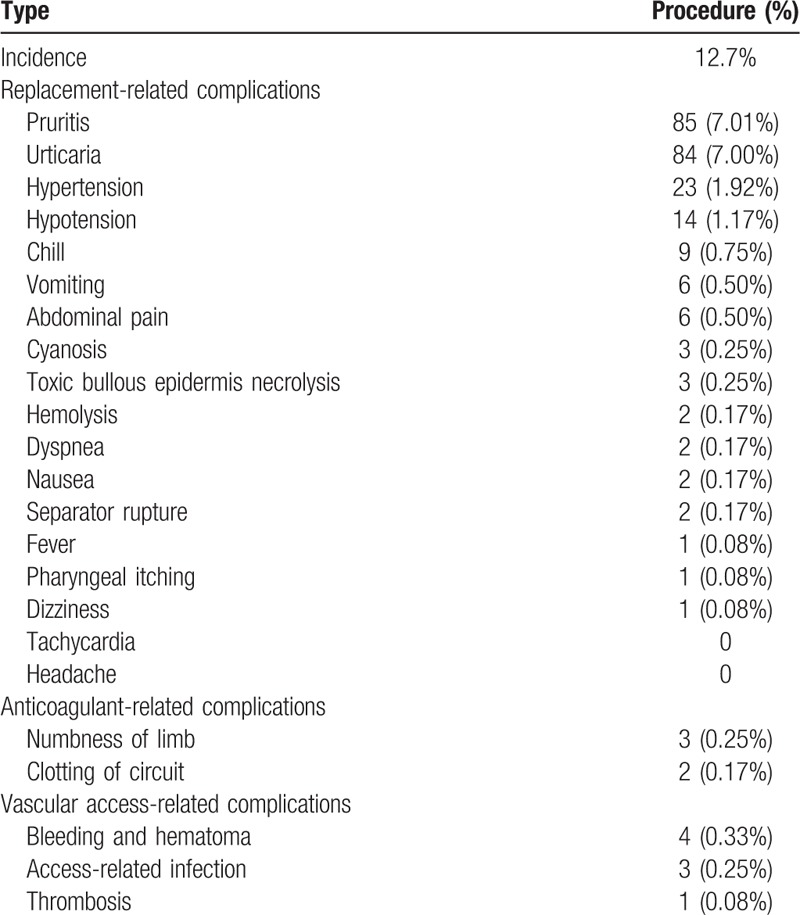
Type of complications of 1201 procedures in 435 children.

Infection occurred in 3 patients. Two patients with anti-*N*-methyl-d-aspartate (anti-NMDA) receptor encephalitis developed catheter-related infection (*Staphylococcus aureus*). One child with SLE developed disseminated cryptococcosis neoformans infection.

Toxic epidermal necrolysis occurred in 3 patients with SLE. They were cured by methylprednisolone (15 mg/kg/day) pulse treatment and hemoperfusion. One child with anti-NMDA receptor encephalitis developed a severe allergic reaction during his 5th TPE treatment, which was relieved by oxygen inhalation, diphenhydramine, and subcutaneous injection of adrenaline.

### 3.4 Complications at different ages and weights

As shown in Table 3, the incidence of complications in children at different ages and weights was significantly different (*P* < .05). The complications were more common in the 6∼15 year group and the 30∼67 kg group. The incidence of pruritus and urticaria in children at different ages and weights was also significantly different (*P* < .05). The incidence of pruritus and urticaria was still more common in the 6∼15 year group and the 30∼67 kg group. However, there was no significant difference in the incidence of hypertension and hypotension between different ages and weights (*P* > .05).

**Table 3:**
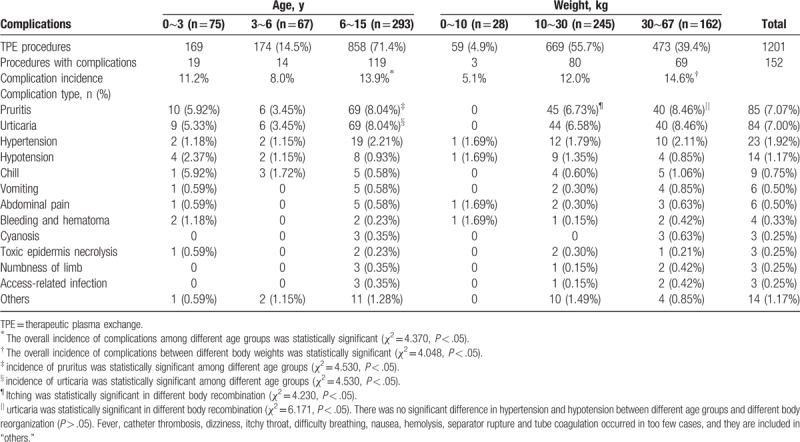
Incidence and type of complications in 435 children at different ages and weights.

### 3.5 Analysis on diagnosis

We selected 5 most common indications (SLE, pesticide poisoning, anti-NMDA receptor encephalitis, rapidly progressive glomerulonephritis, severe purpura nephritis and liver dysfunction) to further analyze the incidence and type of complications. Table 4 shows that the incidence and type of TPE complications were also significantly different between indications. The incidence of complications and the incidence of pruritus and urticaria were the highest in children with anti-NMDA receptor encephalitis (*P* < .05). Complications were rare in patients with other diseases, and thus, no statistical analysis was performed.

**Table 4:**
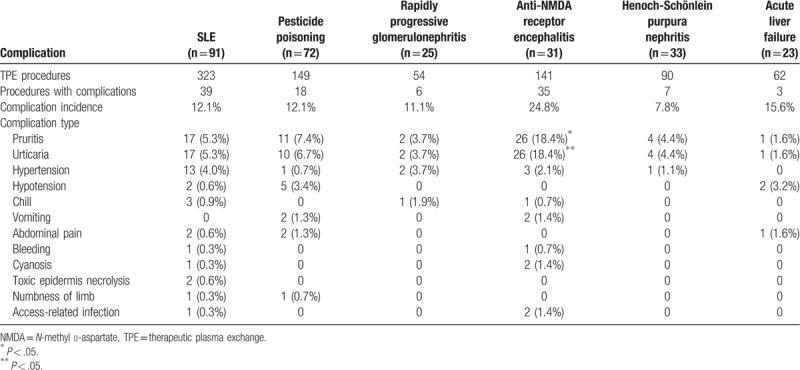
Incidence and type of complications in children with different diseases.

### 3.6 Effect on fibrinogen, albumin, and globulin

Fibrinogen level before treatment (within 1 weeks before the first procedure) and after the last procedure (within 15 days) were collected in 179 children, albumin and globulin in 312 children. Level of fibrinogen and globulin decreased after treatment of therapeutic plasma exchange, whereas albumin shows no significant difference after the treatment (Table 5).

**Table 5:**
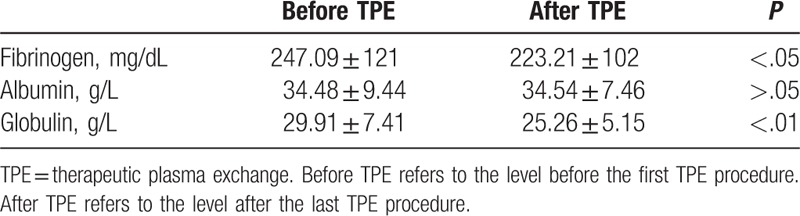
Variation of fibrinogen, albumin, and globulin before and after TPE.

## 4 Discussion

In both adult and child studies, the incidence of TPE complications reported in the literature varies. In 2003, Norda et al^[9]^ reported the registration study of TPE in Sweden. The incidence of TPE complications in 20,485 adults was 4.3% of all procedures. Mokrzycki et al^[5]^ summarized 9 large-scale TPE studies published before 2011. The incidence of complications in adults was 5% to 12% of all procedures, and most of them were <10%.^[10–13]^ However, the incidence of TPE complications in children was 2.2% to 11.0% of all procedures.^[14–19]^ The present study showed that the incidence of TPE complications was 12.7% of all procedures, which is slightly higher than that reported in previous literature. Bambauer et al^[20]^ compared the incidence of TPE complications in patients at different ages. The results showed that the incidence of TPE complications in children <18 years’ old was similar to that in adults aged 18 to 65 years (12.2% vs 9.9%). Witt et al^[21]^ believed that the incidence of TPE complications in children was slightly lower than that in adults (4.0% vs 6.3%). The findings suggest that the incidence of TPE complications in adults and children is similar, and the incidence is not high. In the present study, 75 patients (17.2%) were aged from 3 months to 3 years. Therefore, TPE is even safe to use in infants. Our findings are consistent with those of Fabio et al.^[17]^

We once expected more complication in the lower age group because of their low blood volume and immature organ function,^[16]^ but that is not the fact. The study showed that the incidence of complications in the 6∼15 year group was higher than that in the 0∼3 year and 3∼6 year groups, and the incidence of complications in the 30∼60 kg group was still higher than that in the 0∼10 kg group and 10∼30 kg groups. Older age group appears higher complications, it may be related to higher dosage of plasma, the more plasma is used, the more susceptible to allergy.

The occurrence of TPE complications is influenced by several factors, including anticoagulant methods, types and volumes of replacement fluid, vascular access, diseases, and ways of plasma separation.^[22]^ The present study further analyzed the incidence of complications in patients with different diseases. The results showed that patients with anti-NMDA receptor encephalitis had the highest incidence of complications. Carsten et al reported that the incidence of complications was the highest in patients with neurological diseases.^[13]^ From the results of this study, children with nervous system undergoing plasma exchange should be treated with enhanced monitoring of adverse reactions. However, some studies have shown that the incidence of complications is higher in cryoglobulinemia and thrombocytopenic purpura patients compared with other diseases,^[23,24]^ these findings may be due to selective bias in research subjects, heterogeneous treatment protocols and techniques. There are few cases of bleeding in our study. LMWH was chosen as anticoagulant, as there are ways in which LMWH could be superior to unfractionated heparin: less thrombosis; less bleeding; more convenient, and as well as lower incidence of heparin-induced thrombocytopenia. Besides, it is more convenient in the aspect of monitoring compared to citrate solution. The dosage of LMWH in children is 50 to 80 U/kg and is given to them as the therapy starts.

Common complications include perioral, finger sensory abnormalities caused by hypocalcemia, hypotension, muscle spasm, headache and urticaria related to replacement therapy, establishment of vascular access, and anticoagulation. Most complications of TPE were mild. Basic-Jukic et al^[25]^ reported complications in 509 adult patients with 4857 TPE cases, including abnormal sensation (2.7%), hematoma at the puncture site (2.4%), coagulation (1.7%), and allergic reaction (1.6%). Shemin et al^[23]^ reported complications in 174 adult patients with 1727 TPE, including fever (7.7%), urticaria (7.4%), and hypocalcaemia (7.3%). Rodnitzky et al^[26]^ reported the complications of 17 children with neurological disorders with 154 TPEs, including mild self-limiting toxicity-induced by citrate (7.8%), transient hypotension (2.6%), and amaurosis (1.3%). Gerard et al^[6]^ reported the complications of 18 children with 280 TPEs, including blockage of vascular access (3.6%), bleeding (2.0%), disconnection (1.8%), mild hypotension (1.2%), urticaria (1.2%) and nausea, and vomiting (0.8%). Brunetta et al^[27]^ reported 215 cases of TPE in children, including hypotension (6%), pain or paraesthesia (6%), transfusion reaction (6%), and facial oedema (1%). However, in the present study, the most common complications were pruritus and urticaria (7%), followed by hypertension (1.92%) and hypotension (1.17%).

Previous studies showed that FFP had significantly higher rates of adverse reactions than patients receiving other exchange fluids, such as 5% albumin^[23]^. FFP is associated with the anaphylactoid reaction. Allergic reactions to FFP are characterized by fever, rigors, urticaria, wheezing, and hypotension and can eventually develop into laryngospasm. We do not have large-capacity albumin for plasma exchange in China, and we believe that FFP is more complementary to normal body fluids and immune components than albumin. Therefore, we speculate that the high incidence of pruritus and urticaria is related to the use of FFP in the present study. Although most of the allergic reactions were urticaria and chills, Huestis et al^[28]^ analyzed 42 patients who died of TPE and found that the potential life-threatening complications were still allergic reactions. At least 30 patients died using FFP. In the present study, 2 children suffered from toxic epidermal necrolysis, and 1 child went into anaphylactic shock; all 3 of these patients were forced to discontinue treatment. Therefore, when FFP is used as a replacement fluid, it is necessary to be highly alert to allergic reactions.

The incidence of TPE-induced infection varies greatly, and the pathogenesis of infection is still controversial. Wing et al^[29]^ divided patients with acute glomerulonephritis who received routine treatment (hormones and immunosuppressive agents) into the TPE group and non-TPE group. The results showed that the TPE group had a higher incidence of infection. Aringer et al^[30]^ also found that patients with SLE treated with TPE had an increased risk of severe infection. In the present study, one child with lupus nephritis who received routine treatment (glucocorticoid and immunosuppressants) developed disseminated cryptococcosis neoformans. From the above studies, we cannot exclude the possibility that the removal of immunoglobulins and complement resulted in an immunodeficient state, which impairs the patient's ability to resist infection. Therefore, if severe infection occurs after TPE, the reasonable treatment is to restore normal immunoglobulin levels, which can be achieved by infusing immunoglobulin 100 to 400 mg/kg intravenously.

Previous studies have also shown that TPE may also induce shock, persistent arrhythmia, pulmonary embolism, vascular perforation, severe hemolysis, transfusion-related lung injury, and heparin-induced type II thrombocytopenia.^[31–33]^ Although the above serious complications did not occur in the present study, they should be considered by clinicians and future researchers.

Limitations of the present study include its retrospective design and the fact that it is a single-center analysis. There may be selectivity bias of TPE inclusion criteria and disease type. However, the indications and procedures of TPE treatment in this study are consistent with the recommendations of the American Association for Plasma Separation and Exchange Guidelines in 2016.^[2]^ In addition, the current study is one of the largest single-center cohorts published focused on children, and it adds to the scarce literature on TPE complications in children.

## 5 Conclusions

In conclusion, TPE is a safe blood purification treatment for children. Most of the complications of TPE are mild. The most common complications are pruritus and urticaria. However, serious complications, such as toxic epidermal necrolysis and outbreak of infection, should be considered and prevented to avoid serious consequences.

## Author contributions

**Conceptualization:** Jing Lu, Yuhong Tao.

**Data curation:** Jing Lu, Lijuan Zhang, Cheng Xia, Yuhong Tao.

**Formal analysis:** Yuhong Tao.

**Investigation:** Jing Lu, Lijuan Zhang, Cheng Xia.

**Project administration:** Yuhong Tao.

**Supervision:** Yuhong Tao.

**Writing – original draft:** Jing Lu, Yuhong Tao.

**Writing – review & editing:** Jing Lu, Yuhong Tao.
